# High-fat intake reshapes the circadian transcriptome profile and metabolism in murine meibomian glands

**DOI:** 10.3389/fnut.2023.1146916

**Published:** 2023-03-16

**Authors:** Sen Zou, Jiangman Liu, Hongli Si, Duliurui Huang, Di Qi, Xiaoting Pei, Dingli Lu, Shenzhen Huang, Zhijie Li

**Affiliations:** ^1^Henan Eye Institute, Henan Eye Hospital and Henan Key Laboratory of Ophthalmology and Visual Science, Henan Provincial People’s Hospital, People’s Hospital of Zhengzhou University, People’s Hospital of Henan University, Zhengzhou, China; ^2^Department of Ophthalmology, People’s Hospital of Zhengzhou University, Henan Provincial People’s Hospital, Zhengzhou, China

**Keywords:** bioinformatics, circadian rhythm, high-fat diet, meibomian gland, metabolic dysfunction, RNA-seq, transcriptome

## Abstract

**Background:**

Nutritional and food components reshape the peripheral clock and metabolism. However, whether food challenges affect the circadian clock and metabolism of meibomian glands (MGs) has not been fully explored. This study was designed to analyze alterations in the rhythmic transcriptome and metabolism of MGs of murine fed a balanced diet or a high-fat diet (HFD).

**Methods:**

Male C57BL/6J mice were maintained on a 12/12 h light/dark cycle and fed *ad libitum* on normal chow (NC) or HFD for 4 weeks. MGs were collected from sacrificed animals at 3-h intervals throughout a 24-h circadian cycle. The circadian transcriptome of MGs was analyzed *via* bioinformatics approaches using high-throughput RNA sequencing (RNA-seq). In addition, circadian oscillations of lipid components in MGs were analyzed.

**Results:**

Meibomian glands displayed robust transcriptome rhythmicity. HFD feeding significantly altered the circadian transcriptome profile of MGs—including composition and phase—and spatiotemporally affected the enriched signaling pathways. In addition, HFD feeding significantly altered the normal rhythmic oscillations of lipid components in MGs.

**Conclusion:**

Our data show that HFD significantly affects MGs’ rhythmicity, which reveals a high sensitivity of MGs’ clocks to lipid composition in food.

## 1. Introduction

Meibomian glands (MGs) are sebaceous glands located in the palpebral plate opening at the edge of the eyelid. They provide specialized lipids to the tear film to avoid tear evaporation and overflow and maintain tears between the oily margin and the eyeball to maintain the structural and functional integrity of the ocular surface (1, 2). When the lipids secreted by this gland are altered qualitatively and quantitatively for various reasons, it can result in increased tear evaporation, hyperosmolarity, tear film instability, and bacterial growth at the lid margin, ultimately leading to damage to the ocular surface (3). Currently, MGs dysfunction of various causes is becoming one of the most common diseases in the clinical setting of ophthalmology (3–5). It seriously affects the quality of life of patients. However, our understanding of the structure and physiological function of MGs and the factors affecting them remains extremely limited to date (6).

Given the biological evolutionary drive, the organs, tissues, and physiological processes of any mammalian species can be predicted to undergo significant rhythmic changes accompanying the daily light–dark cycle of the Earth (7–9). Similarly, ocular tissues and their physiological activities undergo synchronous rhythmic changes (10). Published studies, including our team’s series of work, suggest that the cornea (11–13), lacrimal gland (14, 15), retinal pigment epithelium, and retina (16, 17) all exhibit robust rhythmic changes in the phase of the lighting cycle. However, little attention has been paid so far to the circadian rhythmical pattern of MGs and their underlying mechanisms (18, 19). Considering the importance of MGs in maintaining tear film stability through lipid secretion, understanding their circadian rhythmic activity pattern and their associated mechanisms is of clinical importance.

Circadian rhythmicity in mammals shows different patterns, depending on the organ, tissue, and physiological function (7, 20, 21). This rhythmicity is closely coordinated between various organs of the body (22). Many factors, such as high-calorie diets (22) and hypoxia (23), can significantly alter these circadian rhythms and the interconnections between the respective systems. Because of the acceleration of human economic and social activities, a Western diet characterized by high fat content has become prevalent in every corner of the world. Such diets increase the risk of developing many systemic diseases, such as metabolic syndrome, diabetes, and cardiovascular diseases (24). Similarly, the altered composition of high-calorie diets poses a challenge to the physiological function of ocular tissues and the development of disease (25). Metabolic stress from a high-calorie diet can remarkably alter the circadian activity of the cornea and lacrimal gland and the composition of the transcriptome that controls these activities (26). Furthermore, preliminary data suggest that a high-fat diet (HFD) promotes the onset and development of dry eye disease through the induction of an inflammatory response in the lacrimal gland (27, 28). However, the detailed mechanisms are unclear. Therefore, new tools are needed to revisit the HFD-induced dysfunction of MGs and their underlying mechanisms.

Here, we compared the altered transcriptomes of MGs in mice fed a balanced diet and an HFD. Then, the effect of metabolic stress generated by HFD on the circadian clock of MGs and its possible underlying mechanisms were explored by bioinformatics analysis and the detection of diurnal oscillations of lipid droplets in MGs. We found that increased lipid content in food drastically altered the characteristics of the circadian transcriptome of MGs and produced previously unobserved effects on the transcriptome of MGs. This might provide a pathophysiological basis for explaining how food components affect the physiological function of MGs and bring about certain diseases.

## 2. Materials and methods

### 2.1. Animals and dietary interventions

**FIGURE 1:**
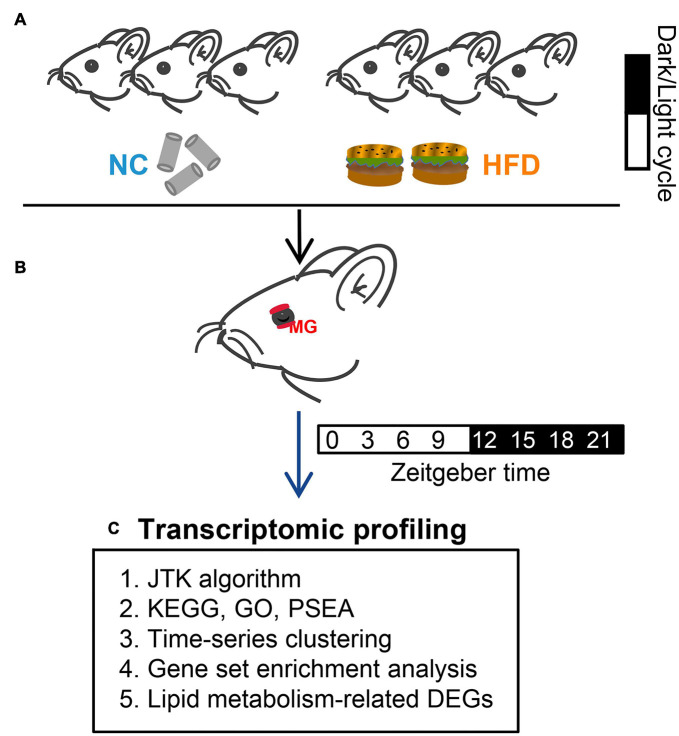
Experimental design. **(A)** After adaption, all mice were divided randomly into two groups. Mice in the NC- and HFD-fed groups were provided with standard normal chow and a high-fat diet for 4 weeks, respectively. **(B)** MGs were obtained from euthanized mice at 3-h intervals (for transcriptomic profiling analysis) or 6-h intervals (for ORO staining) over a 24-h circadian cycle. **(C)** High-throughput sequencing (RNA-Seq) was performed after MG collection. Circadian gene identification and circadian transcriptomic analysis were performed using the Jonckheere–Terpstra–Kendall (JTK) cycling algorithm. The biological processes and molecular function of genes were annotated by the Kyoto Encyclopedia of Genes and Genomes (KEGG), Gene Ontology (GO), phase set enhanced analysis (PSEA), time-series clustering analysis, and gene set enriched analysis (GSEA).

Six-week-old male C57BL/6J mice were obtained from Nanjing University in China and housed in light-tight circadian chambers (12/12 h light/dark daily cycle) (Longer-Biotech Co., Ltd, Guangzhou, China) (29). The Zeitgeber time (ZT) scale was used here to record the time: ZT0 referred to time of lights on (7 a.m.), and ZT12 referred to lights off (7 p.m.) (30). Mice were provided with *ad libitum* access to their respective diets throughout the study. After 2 weeks of adaption in light-tight circadian chambers, all mice (8 weeks old now) were divided randomly into two groups. The normal chow (NC) group mice were provided with standard NC with 9% kcal fat (Trophic Animal Feed High-Tech Co., Ltd., Nantong, China) for 4 weeks. The HFD group mice were provided with HFD with 60% kcal fat (Trophic Animal Feed High-Tech Co., Ltd., Nantong, China) for 4 weeks (Figure 1A), as previously described (26). RNA-Seq data for circadian analysis were collected at eight time points throughout the circadian cycle (3-h intervals) (Figure 1B). Circadian gene identification and circadian transcriptomic analysis (phase and amplitude) were performed by the Jonckheere–Terpstra–Kendall (JTK) cycling algorithm. The biological processes and molecular function of genes were annotated by the Kyoto Encyclopedia of Genes and Genomes (KEGG), gene ontology (GO), phase set enhanced analysis (PSEA), time-series clustering analysis, and gene set enriched analysis (GSEA) (Figure 1C). Circadian changes in lipid droplets in MGs were studied by Oil Red O (ORO) staining. All mice were euthanized by cervical dislocation after inhalation of ether.

### 2.2. MG collection, total RNA extraction, and RNA-seq

After exposure to NC or HFD dietary regimens, the upper and lower MGs from the left eyelid were collected and combined from euthanized animals at 3-h intervals over the circadian cycle from NC- and HFD-fed mice, as previously described (27, 31). Total RNA was isolated from the MGs using an RNAeasy spin column kit (Qiagen). For each ZT point, RNA-Seq analysis was performed using three biological replicates (15, 30). Library preparation and sequencing for the total RNA of MGs were performed according to our previous report (26, 30, 32). In brief, total RNA was quantified using a NanoDrop spectrophotometer (Thermo Fisher Scientific, MA, USA). The cDNA was amplified by PCR, and raw reads were filtered by SOAPnuke (Version v1.5.2) (33). HISAT2 (34) and Bowtie2 were used to align the clean reads (reference: Mus_musculus, GCF_000001635.26_GRCm38. p6) (35). Differentially expressed genes (DEGs) between the NC- and HFD-fed groups were identified using the R software edgeR package.^1^

### 2.3. Analysis of rhythmic genes

The circadian genes of MGs were identified using the JTK_CYCLE algorithm in R software, as previously described (26, 30, 32). The time-ordered fragments per kilobase of exon model per million mapped fragments (FPKM) of all MG genes were imported into the algorithm. Rhythmic genes with a period of 24 h were identified, and the phases with amplitudes of the rhythmic genes were also determined. All MG genes were composed of low expression genes (FPKM < 0.1), rhythmic genes (FPKM ≥ 0.1 and Bonferroni-adjusted *P* < 0.05), and non-rhythmic genes (FPKM ≥ 0.1 and Bonferroni-adjusted *P* ≥ 0.05).

### 2.4. Functional annotation by KEGG, GO, PSEA, and GSEA

Biological processes and molecular function annotations of MG genes were performed using KEGG, GO, PSEA, and GSEA, as previously described (26, 30, 32). KEGG and GO enrichment analysis were performed by online bioinformatic platform Dr. Tom,^2^ an online software developed by Beijing Genomic Institute (BGI) (36). PSEA software (v1.1) was used to annotate rhythmic genes at the oscillating phase’s level with the reference gene set (c2.cp.kegg.v7.2.symbols.gmt) downloaded from MSigDB^3^ (30). GSEA software (v3.0) was used to characterize the biological pathways of MG genes by reference gene sets c5.go.bp.v7.2.symbols.gmt and c2.cp.v7.2.symbols.gmt. The significance threshold of the *Q*- or *P*-value for the analysis was 0.05 (26, 30, 32).

### 2.5. Time-series clustering analysis and protein–Protein association networks

To reveal dynamic expression trends in the rhythmic genes of the MGs, the fuzzy c-means clustering algorithm in the Mfuzz package was adopted, as previously described (30). In this paper, the number of clusters in the rhythmic genes of NC- and HFD-fed mice was set as 4 on the basis of gene expression trends, with default values for other parameters. To visualize the gene--gene interaction of lipid--metabolic genes in the NC- and HFD-fed mice, protein--protein association network (PPAN) analysis was performed *via* STRING analysis.^4^ The parameters in the full STRING network were as follows: meaning of network edges, evidence; active interaction sources, experiments and databases, kmeans clustering method with 3 as the number of clusters.

### 2.6. Immunohistochemistry of MGs

After dietary intervention, eyelid tissues with eyeballs were collected from the right side of the NC- and HFD-fed mice at 6-h intervals throughout a 24-h circadian cycle (ZT0, 6, 12, 18), as previously described (15, 26). In brief, paraffin tissues were collected for hematoxylin and eosin staining to visualize the morphology of the MG tissues, and frozen sections were prepared for ORO staining (G1016, Servicebio Company). Eyelid tissues were cut into sagittal sections (5 μm thick). MG sections were immersed in ORO solution for 10 min in the dark. ORO staining was analyzed by mean optical density using ImageJ software (version 1.42q; National Institutes of Health, USA). Representative ORO staining images of the NC- and HFD-fed MGs were selected using CaseViewer software (3DHISTECH Ltd., Budapest, Hungary).

### 2.7. Statistical analysis and software

Statistical analysis and figure preparation were processed using GraphPad Prism 9.3.1. A heatmap of circadian genes was prepared using the “*pheatmap*” package in R software. Data with normal distribution were statistically analyzed using the Student’s *t*-test to compare the differences between the NC- and HFD-fed mice. A value of *P* < 0.05 indicated a statistically significant difference.

## 3. Results

### 3.1. HFD alters the characteristics of circadian transcriptome in murine MGs

**FIGURE 2:**
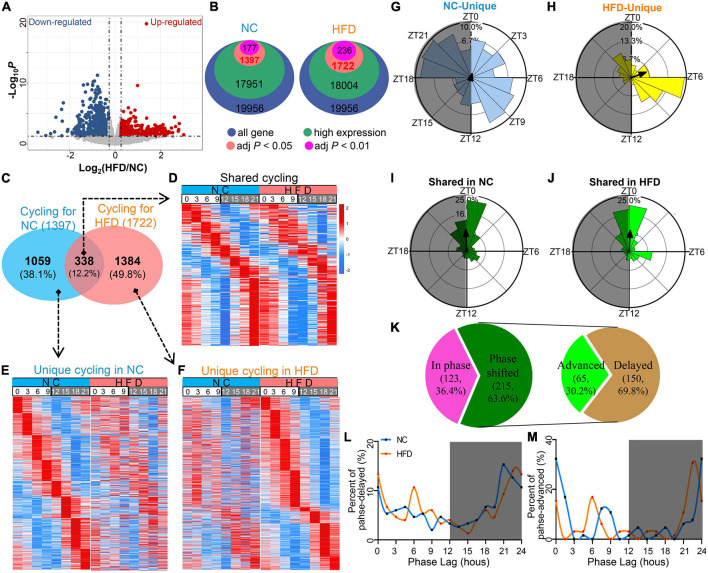
High-fat diet (HFD) reprograms the characteristics of the circadian transcriptome in murine MGs. **(A)** Volcano plot of RNA-seq data for NC- and HFD-fed MG genes. The red and blue dots denote ≥1.2-fold or ≤0.83-fold changes in expression between the NC-fed and HFD-fed MGs, respectively. **(B)** The number of rhythmic genes in NC- and HFD-fed MGs under different threshold conditions in the JTK_ algorithm. **(C)** Venn diagram showing the gene sets of the MGs of NC- and HFD-fed mice (JTK algorithm, adjusted *P* < 0.05 and expression ≥ 0.1). *n* = 3 mice per group per time point at 3-h intervals. *n* = 24 mice per group. **(D)** Heatmaps visualizing the expression levels of 338 shared rhythmic genes in the MGs of NC- (left) and HFD-fed (right) mice at different ZT points at 3-h intervals throughout the circadian cycle. The expression levels were indicated by a color bar ranging from blue to red, with the expression range normalized to ± 2. **(E)** Heatmaps visualizing the expression levels of 1,059 rhythmic genes unique in the MGs of NC-fed mice at various ZT points at 3-h intervals throughout the circadian cycle. **(F)** Heatmaps visualizing the expression levels of 1,384 rhythmic genes unique in the MGs of HFD-fed mice at various ZT points at 3-h intervals throughout the circadian cycle. **(G–J)** Phase analysis of rhythmic genes in the MGs of NC- and HFD-fed mice. Gray shading: dark phase. **(K)** Phase analysis of 338 shared rhythmic genes in the MGs of NC- and HFD-fed mice. Phase distribution plot for phase-delayed **(L)** and phase-advanced genes **(M)** in shared cycling genes. Gray shading: dark phase.

To visualize the transcriptomic differences between NC- and HFD-fed MGs, we performed a comparative expression analysis of RNA-seq data using a volcano plot (Figure 2A). We identified 1,397 and 1,722 circadian genes (Supplementary Table 1, JTK_adj *P* < 0.05) from all the MG genes of the NC- and HFD-fed mice, respectively (Figures 2B, C). In total, 338 cycling genes were shared between the two diet interventions; 1,059 were unique to the NC-fed MGs, and 1,384 were unique to the HFD-fed MGs (Figure 2C and Supplementary Table 2). HFD intervention did not significantly alter the oscillation patterns of shared rhythm genes in MGs within 24 h at 3-h intervals (Figure 2D). The peak expression of NC-unique cycling genes in MGs was throughout the circadian cycle, but they did not show a circadian rhythmic expression pattern in HFD-fed MGs (Figure 2E). In contrast, HFD-unique cycling genes were mainly expressed in the light phase, whereas they did not show a circadian rhythmic pattern in NC MGs (Figure 2F).

The expression phase of NC-unique rhythmic genes was mainly in ZT6 to ZT10.5 and ZT18 to ZT22.5 and throughout the circadian cycle (Figure 2G). Importantly, the phase of HFD-unique rhythmic genes peaked in ZT6 to ZT9 (Figure 2H). In contrast, the shared rhythmic genes were mainly from ZT0 to ZT1.5 in the NC-treated MGs (Figure 2I) and from ZT22.5 to ZT1.5 in the HFD-treated MGs (Figure 2J). For the shared cycling genes, 63.6% were phase shifted, whereas 36.4% were in phase (Figure 2K). Of the phase-shifted cycling genes, 30.2% were advanced in phase, and 69.8% were delayed (Figures 2K–M). There was no significant difference in the amplitude of cycling genes in shared or unique cycling genes between the MGs of NC- and HFD-fed mice (Supplementary Figure 1). Collectively, these data suggest that, under homeostatic conditions, HFD intervention dramatically altered the composition, number, and oscillation phase of rhythm genes in murine MGs.

### 3.2. HFD alters the functional characteristics of cycling genes in mouse MGs

**FIGURE 3:**
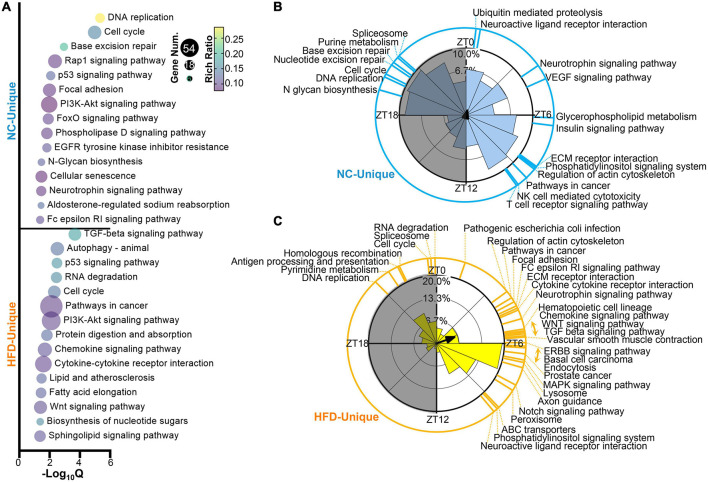
High-fat diet (HFD) alters the oscillatory characteristics of circadian transcriptomic profiling in murine MGs. **(A)** Functional annotations for NC-unique (up) and HFD-unique (down) cycling genes by GO Biological Process analysis (*Q* < 0.05). Phase distribution of significantly enriched KEGG pathways (*Q* < 0.05) in the MGs of NC-fed **(B)** and HFD-fed **(C)** mice. The blue **(B)** and yellow **(C)** lines on the outside circle indicate the enriched pathways at various phases. Gray shading: dark phase.

To evaluate the effect of HFD feeding on the biological processes of cycling genes, we performed GO annotations for MG genes in NC- and HFD-fed mice. The NC- and HFD-specific cycling genes were enriched in various biological processes, especially in the immune, metabolic, and nervous systems, as shown in Figure 3A. PSEA analyzes were performed to characterize the effect of HFD intervention on the spatiotemporal distribution of the signaling pathways of cycling genes. The pathways enriched in the NC-fed MGs were distributed throughout the circadian cycle, whereas those in the HFD-fed group were mainly located in the light phase (Figures 3B, C). Importantly, more immune-related pathways were enriched in the MGs of NC- fed mice, and more important signaling pathways were enriched in the light phase of the MGs of HFD-fed mice (Figures 3B, C). In summary, our results indicate that HFD intervention significantly rewired the rhythmic activity in the GO and PSEA levels, which may result in changes in the potential functions of these rhythmic genes in the MGs of HFD-fed mice.

### 3.3. HFD alters the cluster-dependent transcriptomic map

**FIGURE 4:**
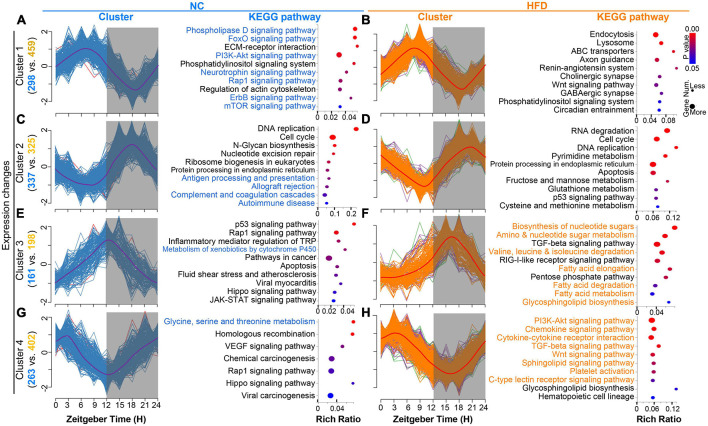
High-fat diet (HFD) alters the cluster-dependent transcriptomic map. **(A,C,E,G)** Oscillating patterns of normalized expression for rhythmic genes from four enriched clusters for the MGs of NC-fed mice (left). The enriched KEGG pathways for genes in each cluster (*P* < 0.05) are shown in the (right) panel. Gray shading: dark phase. **(B,D,F,H)** Oscillating patterns of normalized expression for rhythmic genes from four enriched clusters for the MGs of HFD-fed mice (left). The enriched KEGG pathways for genes in each cluster (*P* < 0.05) are shown in the (right) panel. Gray shading: dark phase.

To reveal the dynamic expression trends in the rhythmic genes of the MGs after HFD intervention, we analyzed the time series clustering analysis of cycling genes in the MGs of NC- and HFD-fed mice. Four oscillating patterns were determined on the basis of the positions of the peaks and troughs in the NC or HFD groups. The peaks of Cluster 1 were located at ZT6 and the troughs at ZT18, and the 298 and 459 cycling genes were enriched in the MGs of NC- and HFD-fed mice, respectively (Figures 4A, B). The peaks of Cluster 2 were located at ZT18 and the troughs at ZT6, and 337 and 325 cycling genes were enriched in the MGs of NC- and HFD-fed mice, respectively (Figures 4C, D). The peaks of Cluster 3 were located at ZT12 and the troughs at ZT0, and 161 and 198 cycling genes were enriched in the MGs of NC- and HFD-fed mice, respectively (Figures 4E, F). The peaks of Cluster 4 were located at ZT3 and the troughs at ZT15, and 263 and 402 cycling genes were clustered in the MGs of NC- and HFD-fed mice, respectively (Figures 4G, H). Cycling genes in each cluster of the MGs of NC- and HFD-fed mice are listed in Supplementary Table 3.

The KEGG annotation functions for cycling genes with similar temporal patterns between the MGs of NC- and HFD-fed mice had significantly different annotation pathways (Figures 4A–H, right of each panel). Rhythmic genes in cluster 2 of the MGs of NC-fed mice were enriched mainly in immune function (Figure 4C), whereas the cluster 4 genes of the MGs of HFD-fed mice were associated with immune pathways (Figure 4H). The cycling genes of cluster 1 in the MGs of NC-fed mice were mainly related to important signaling pathways (Figure 4A), whereas similar pathways in the MGs of HFD-fed mice were concentrated in Cluster 4 (Figure 4H). Cluster 3 genes in the MGs of HFD-fed mice were related to metabolism pathways, especially fat metabolism (Figure 4F), whereas a few pathways were associated with metabolism function in the MGs of NC-fed mice (Figure 4). Collectively, these results suggest that HFD intervention reshapes the oscillating patterns and corresponding functional pathways of rhythmic genes.

### 3.4. HFD does not elicit core clock desynchrony of MGs

**FIGURE 5:**
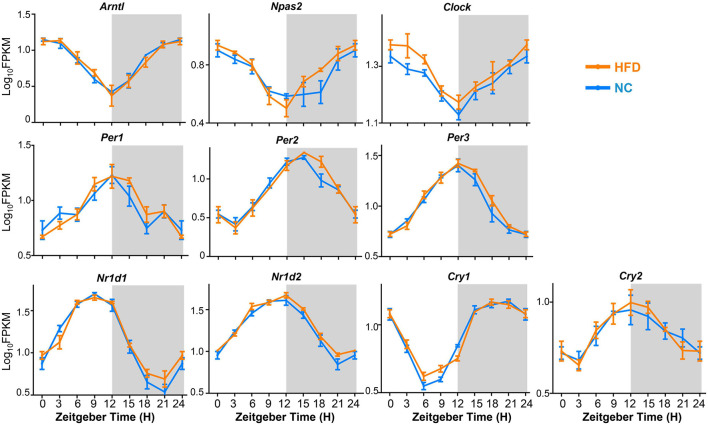
Expression and oscillation patterns of core clock machinery genes in the MGs of NC- and HFD-fed mice. *n* = 3 mice per group for each sampling time point. Student’s *t*-test was performed for each ZT time point for the NC- and HFD-fed mice. Gray shading: dark phase.

To determine the effect of HFD intervention on the oscillatory pattern of core clock machinery genes in the mouse MG, we compared the expression levels and oscillation amplitudes of the core clock genes, including *Arntl* (*Bmal1*), *Npas2*, *Clock*, *Per1*, *Per2*, *Per3*, *Nr1d1*, *Nr1d2*, *Cry1*, and *Cry2*, between the MGs of NC- and HFD-fed mice at 3-h intervals over a 24- h circadian cycle. The results showed that the expression of all these core clock genes exhibited significant diurnal rhythmicity in MGs from NC- and HFD-fed mice (Figure 5). However, the phase distribution and oscillation amplitude of core clock gene expression were not significantly altered in the MGs of HFD-fed mice compared to those of NC-fed mice (Figure 5). Thus, these data suggest that HFD intervention does not interfere with the synchronization of the core clock machinery in MGs.

### 3.5. HFD-induced lipid metabolism disorder in MGs

**FIGURE 6:**
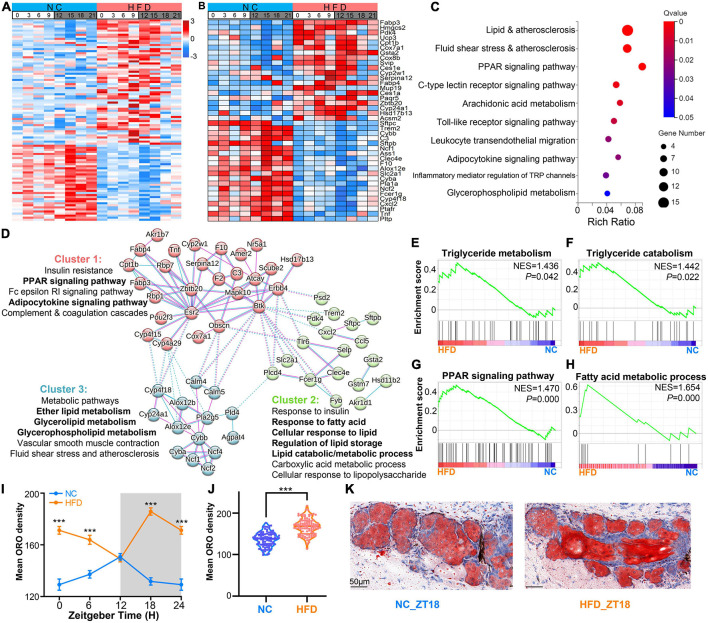
High-fat diet (HFD)-induced lipid metabolism disorder in MGs. **(A)** Heatmaps visualizing the expression levels of the differentially expressed lipid-associated genes (fold change ≥1.2 or ≤0.83, adjust *P* < 0.05) in MGs between the NC- and HFD-fed mice at various ZT points at 3-h intervals throughout the circadian cycle. The expression levels were indicated by a color bar ranging from blue to red, with the expression range normalized to ± 3. **(B)** Heatmaps visualizing the expression levels of the top 20 up- and down-regulated DEGs of lipid metabolism-related genes in the MGs between the NC- and HFD-fed mice at various ZT points. **(C)** The top 10 significant KEGG annotations of 98 DGEs associated with lipid metabolism in the MGs between the NC- and HFD-fed mice (*Q* < 0.05). **(D)** The protein–protein association networks (PPANs) and functional clusters with specific KEGG annotations of lipid metabolism-related DEGs in the MGs between the NC- and HFD-fed mice (*Q* < 0.05). **(E–H)** Enrichment plots for triglyceride metabolism/catabolism, PPAR signaling pathway, and fatty acid metabolic process were enriched specifically in the MGs of HFD-fed mice by GSEA analysis. **(I)** Temporal changes in lipid droplets in the MGs of NC- and HFD-fed mice at 6 -h intervals. Three to five right-sided MGs were randomly selected from each NC- and HFD-fed mouse. *n* = 6 mice per group per sampling time point. Student’s *t*-test was performed for each ZT point in the NC- and HFD-fed mice. ***P < 0.001. The gray shading indicates the dark phase. **(J)** Average lipid droplet accumulation in the MGs of NC- and HFD-fed mice. *n* = 24 mice per group. Student’s *t*-test between the NC- and HFD-fed mice. ***P < 0.001. **(K)** Representative ORO staining images of lipid deposition in the MGs of NC- (left) and HFD-fed (right) mice at ZT18. Scale bar: 50 μm.

To verify the effects of HFD intervention on the lipid metabolism-related genes and their potential functions in murine MGs, we compared the differential expression level of genes between the MGs of NC- and HFD-fed mice (fold change ≥1.2 or ≤0.83, adjust *P* < 0.05). As shown in Figure 6A and Supplementary Table 4, 98 DEGs related to lipid metabolism were found, of which 61 were upregulated in the MGs of HFD-fed mice, and 37 genes were downregulated. The top 20 up- and down-regulated DEGs at various ZT points are shown in Figure 6B. The DEGs related to lipid metabolism were enriched in some lipid metabolism pathways (*Q* < 0.05), as shown in Figure 6C. Analyzes by PPANs (Figure 6D) and GSEA (Figures 6E–H) were performed to investigate the enrichment of genes in specific molecular functions. These data revealed that significantly enriched signaling pathways were related to specific lipid metabolism, including glycerolipid/glycerophospholipid/ether lipid metabolism, response to lipid/fatty acid, regulation of lipid storage, and lipid catabolic/metabolic process (Figure 6D). The GSEA results revealed that triglyceride metabolism/catabolism, PPAR signaling pathway, and fatty acid metabolic process were enriched specifically in the MGs of HFD-fed mice (Figures 6E–H).

To determine the effect of HFD feeding on lipid metabolism in MGs, we performed ORO staining to observe the differences in lipid droplets between the MGs of NC- and HFD-fed mice. The results showed that lipid amounts showed a strong rhythm in the MGs of NC-fed mice, with lipid droplets peaking at ZT12 and troughing at ZT0 (Figure 6I). In contrast, in the MGs of HFD-fed mice, lipid amounts peaked at ZT18 and trough at ZT12 (Figure 6I). In addition, the amount of lipids in the MGs of HFD-fed mice was significantly higher than that in the MGs of NC-fed mice (Figures 6J, K). These results suggest that HFD intervention alters lipid metabolism in murine MGs and causes lipid accumulation in MGs.

## 4. Discussion

To the best of our knowledge, this is the first study to show that high-fat nutritional stress uniquely affects the circadian transcriptome of murine MGs. We found that a 4 weeks high-fat dietary regimen significantly altered the circadian characteristics of MGs, including their cycling transcriptome profiles and content of lipid droplets. Notably, high-fat intake shifts cycling genes and their enriched functional signaling pathways that occur throughout the light–dark cycle in the MGs of balanced diet-fed mice to only the light phase of HFD-fed mice. These data suggest that the nutritional challenges posed by short-term, high-fat dietary intake reorganize the circadian rhythms of MGs.

In mammals, circadian physiology is generated or controlled by the suprachiasmatic nucleus (SCN), a central pacemaker in the hypothalamus (37, 38). The SCN generates or controls output circadian physiology through diffusible signals, including hormonal rhythms, sympathetic/parasympathetic systems, core body temperature, and feeding patterns, to control the molecular clock in peripheral tissue cells, thereby generating output circadian physiological activity (8). However, many exogenic zeitgebers (39–41), including nutritional alternations (42–44), feeding timing (29, 45), and altered sleep/wakefulness (46) can disrupt the normal circadian rhythm and promote the occurrence of some diseases, such as metabolic syndrome and type 2 diabetes (47, 48).

Previous studies by us and other teams have found that interventions, such as short-term HFD (26), high fructose intake (32), jet lag (15), and gut dysbiosis accompanying aging (14), can reformat the rhythmic profile of the murine lacrimal gland. Similarly, a high fructose intake significantly alters the rhythmic pattern of the murine corneal transcriptome and its associated physiological activities. Consistent with these studies, the present study confirms that a 4 weeks high-fat dietary regimen reshapes the composition of rhythmic transcriptome and their functional signaling pathways enriched in mouse MGs at a spatiotemporal level. These results suggest that the nutritional challenge from an HFD alters the circadian rhythmicity of MGs in a tissue-specific manner. Therefore, further exploration of the underlying mechanisms will likely be of high significance.

Each mammalian cell contains a machinery of core clock genes that generate rhythmic oscillatory gene expression and its associated physiological activities in a 24-h cycle by binding thousands of pathways to the entire genome and driving a feedback regulatory system (49). Core clock genes are the central controllers of the biological clock system. The available data suggest that the core clock system of the cell is a relatively stable system. If the retino-hypothalamic tract (RHT) system is not disturbed, the core clock system maintains a steady state (50, 51). This stability is not only present in metabolic stress but also in aging organs and tissues (51–53). Similarly, the same stabilization phenomenon has been observed in ocular tissues subjected to nutritional challenges, such as the cornea (11) and the lacrimal gland (26) subjected to high fructose intake and the lacrimal gland subjected to a high-fat diet (26). Similarly, this asynchrony is present in the nutritionally challenged liver (54), as well as in several aging tissues (51–53). Recently, we found that the core clock of the lacrimal gland was not significantly altered, even in sleep deprivation-treated mice, without altering the light/dark cycle, although the output gene fraction was drastically altered (55). These studies further confirm the strong stability of the core clock without altering the day/night cycle. However, the cause of the altered output genes under the aforementioned nutritional stress and other factors has thus far been unclear. Recently, Deota et al. speculated that the rhythmicity of output gene expression in most tissues is not exclusively driven by the circadian clock. Systemic signals generated by other factors (e.g., feeding-fasting cycles) combined with endogenous clock modulated signals may play a dominant role in regulating the rhythmicity of gene expression in peripheral organs (56). Therefore, further exploration of the mechanisms by which HFD leads to decoupling the core clock from the downstream core clock-controlled output system is potentially valuable for addressing the pathophysiological alterations in the structure and function of MGs caused by HFD.

High-throughput RNA-seq data-based bioinformatics analysis is currently one of the main tools used to elucidate the complex molecular mechanisms behind circadian rhythm alterations. Time series clustering methods provide an effective approach for assessing the accompanying temporal features for big data analysis (57). The present study provides another dimensional analysis of the pattern of altered physiological activity of MGs due to excessive lipid intake. Consistent with previously studied ocular tissues, such as the cornea (11) and lacrimal gland (26), nutritional challenges dramatically altered the output transcriptome of circadian rhythms in both gene composition and the oscillations of their enriched signaling pathways. MGs play an important role in maintaining the stability of the tear film mainly through the lipid layer of the tear film (3, 58, 59). Therefore, we specifically analyzed the effect of HFD on the lipid metabolism-related transcriptome of MGs and the content of lipid droplets accompanying temporal oscillations. As predicted, this study confirms that HFD has a profound effect on lipid metabolism-related pathways and oscillations of lipid droplets in MGs. These data provide new insights into HFD-induced MG dysfunction. However, further exact mechanisms would require further in-depth analysis by lipidomics, proteomics, and metabolomics.

This study has several limitations. First, the C57BL/6 mice used in this study were nocturnal animals. The sleep–wake cycle in mice is opposite to that in humans (60). Therefore, certain facts about human MGs must be interpreted with caution. Second, the present study only provided changes in the transcriptomic profile of MGs in male mice, and further observations in female mice may provide more information, especially regarding sex-specific differences (61, 62). Third, the C57BL/6 mice used in this study are melatonin-deficient mouse models (63), but melatonin plays an important role in regulating circadian rhythms in humans (64). Therefore, not all phenomena that occur in humans can be addressed. Fourth, this paper focuses on the bioinformatic interpretation of the effect of HFD on the transcriptomic rhythmicity of MGs, and we will attempt to focus more on the cellular and molecular mechanisms in future studies. Finally, this project provides only the effect of HFD on the bulk transcriptome rhythmicity of MGs. Considering the complexity of the different cell types of MGs and their existence of different oscillatory cycles, the use of single-cell RNA-seq sequencing technology in the future will offer solutions to this problem (65, 66).

## 5. Conclusion

**FIGURE 7:**
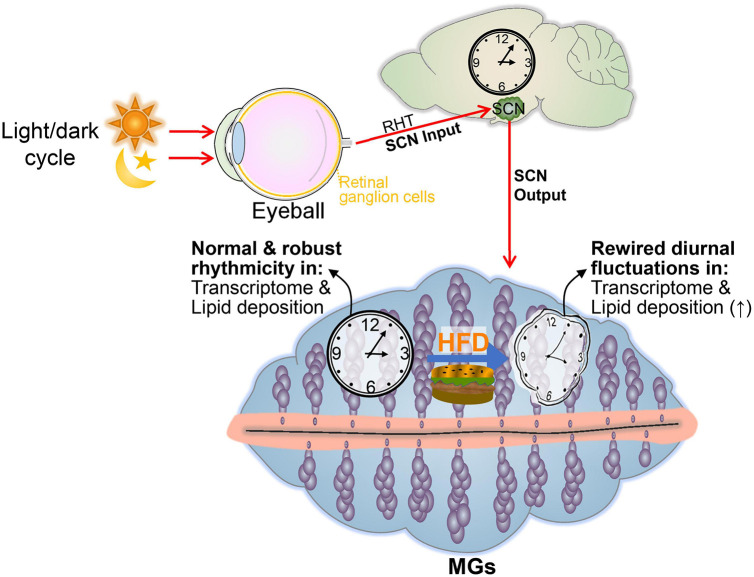
Summary displaying the effects of an HFD on the cyclical transcriptomic profile of MGs. In mice receiving a high-fat dietary regimen, the light-regulated central clock pacemaker (SCN) functions normally and expresses normal sleep/wake and fasting/feeding rhythms. However, a high-fat diet alters the normal circadian rhythmicity transcriptome profiles and lipid droplet oscillation of MGs.

In conclusion, our observations support the concept that an HFD alters the output component of the circadian rhythm of the MGs, rather than the core clock machinery (Figure 7). These data emphasize the importance of nutritional interventions in maintaining the health of MGs. Exploring or targeting the loss-of-coupling mechanism between the core clock and the output component has the potential to ameliorate MG dysfunction induced by HFD.

## Data availability statement

The original contributions presented in this study are publicly available. This data can be found here: https://www.ncbi.nlm.nih.gov/bioproject/PRJNA924579.

## Ethics statement

All animal experiments in this study were approved by the Animal Ethics Committee of Henan Provincial People’s Hospital and followed the guidelines described in the ARVO Statement for the Use of Animals in Vision and Ophthalmic Research.

## Author contributions

ZL and SZ designed the study and wrote the manuscript. SZ, ZL, JL, HS, DH, and DQ collected and prepared the samples. SZ performed RNA-seq and bioinformatics analysis with help from XP, DL, and SH. All authors contributed to the article and approved the submitted version.

## Footnotes

1
https://bioconductor.org/packages/release/bioc/html/edgeR.html

2
biosys.bgi.com/

3
https://www.gsea-msigdb.org/gsea/msigdb/

4
https://string-db.org/

## Supplementary material

Supplementary Figure 1 Amplitudes (AMP) of shared **(up)** and unique **(down)** rhythmic genes in the MGs of NC- and HFD-fed mice. Student’s *t*-test between NC- and HFD-fed mice. ns, not significant.

Supplementary Table 1 Cycling genes (JTK_adj *P* < 0.05) of all MG genes for NC- (1,397) and HFD-fed (1,722) mice in Figure 2B.

Supplementary Table 2 Cycling genes (JTK_adj *P* < 0.05) unique to NC-fed (1,059), unique to HFD-fed (1,384) and shared between the two groups (338) in Figure 2C.

Supplementary Table 3 Rhythmic genes in each time-series cluster of NC- and HFD-fed MGs in Figure 4.

Supplementary Table 4 Expression levels of lipid-related DEGs for NC- and HFD-fed MGs in Figure 6A.
