# Supplementary figures and images for: High-fat intake reshapes the circadian transcriptome profile and metabolism in murine meibomian glands
Source: Front Nutr. 2023 Mar 16;10:1146916. doi: 10.3389/fnut.2023.1146916 (PMC10062204; doi:10.3389/fnut.2023.1146916)

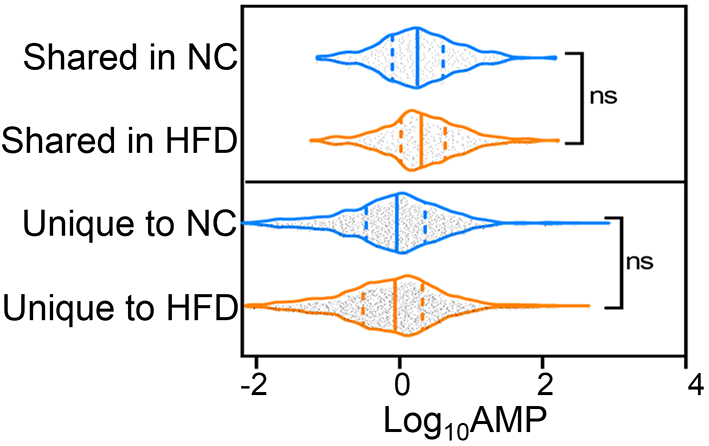

Supplement: Supplementary Figure 1 — Amplitudes (AMP) of shared (up) and unique (down) rhythmic genes in the MGs of NC- and HFD-fed mice. Student’s t-test between NC- and HFD-fed mice. ns, not significant. [file Image_1.TIF]
